# Mitigative Effect of Erythromycin on PMMA Challenged Preosteoblastic MC3T3-E1 Cells

**DOI:** 10.1155/2014/107196

**Published:** 2014-06-03

**Authors:** Yi Shen, Weili Wang, Xiaomiao Li, David C. Markel, Weiping Ren

**Affiliations:** ^1^Department of Orthopaedic, Ren Ji Hospital, School of Medicine, Shanghai Jiao Tong University, 1630 Dongfang Road, Shanghai 200127, China; ^2^Department of Orthopedic Surgery, Providence Hospital & Detroit Medical Center Orthopedic Residency, Detroit, MI 48201, USA; ^3^Department of Biomedical Engineering, Wayne State University School of Medicine, 818 W. Hancock, Detroit, MI 48201, USA

## Abstract

*Background*. Aseptic loosening (AL) is a major complication of total joint replacement. Recent approaches to limiting AL have focused on inhibiting periprosthetic inflammation and osteoclastogenesis. *Questions/Purposes*. The purpose of this study was to determine the effects of erythromycin (EM) on polymethylmethacrylate (PMMA) particle-challenged MC3T3 osteoblast precursor cells. *Methods*. MC3T3 cells were pretreated with EM (0–10 *μ*g/mL) and then stimulated with PMMA (1 mg/mL). Cell viability was evaluated by both a lactate dehydrogenase (LDH) release assay and cell counts. Cell differentiation was determined by activity of alkaline phosphatase (ALP). Gene expression was measured via real-time quantitative RT-PCR. *Results*. We found that exposure to PMMA particles reduced cellular viability and osteogenetic potential in MC3T3 cell line. EM treatment mitigated the effects of PMMA particles on the proliferation, viability and differentiation of MC3T3 cells. PMMA decreased the gene expression of Runx2, osterix and osteocalcin, which can be partially restored by EM treatment. Furthermore, EM suppressed PMMA- induced increase of NF-*κ*B gene expression. *Conclusions*. These data demonstrate that EM mitigates the effects of PMMA on MC3T3 cell viability and differentiation, in part through downregulation of NF-*κ*B pathway. EM appeared to represent an anabolic agent on MC3T3 cells challenged with PMMA particles.

## 1. Introduction

Aseptic loosening (AL) is a major complication of total joint replacement (TJR) [1]. AL primarily results from a biological response of inflammatory cells and osteoblasts to wear particles [2, 3]. Recent approaches to limit osteolysis have focused on inhibiting periprosthetic inflammation and osteoclastogenesis [4, 5].

Erythromycin (EM), a 14-membered lactone ring macrolide antibiotic, has been used for infectious disease for more than 50 years. EM has additional anti-inflammatory effects far beyond antibiotics [6]. An example of EM used as an anti-inflammation drug has been demonstrated previously in Japan, where EM was successful in treating diffuse panbronchiolitis (DPB), a noninfectious inflammatory lung disease of unknown cause [7]. There is compelling evidence that EM exerts its anti-inflammatory effects through targeting NF-*κ*B signaling [8, 9]. We propose that EM is a promising drug candidate for AL because of its unique property of favorably concentrating in bone marrow and inflammatory cells, especially in monocyte/macrophages [10]. We have completed a series of experiments to test our hypothesis. First, we found that EM inhibits wear debris-induced osteoclastogenesis by inhibition of NF-*κ*B activity in both a RAW264.7 macrophage cell line and mouse bone marrow progenitor cells [9]. Second, we found that EM inhibits wear particle-induced inflammatory osteolysis in a murine osteolysis model [11]. Third, encouraged by these laboratory findings, we designed a prospective clinical trial and revealed that oral EM (600 mg/daily) inhibits periprosthetic tissue inflammation in 32 AL patients who were candidates for surgical revision [12]. Taken together, EM represents an appropriate drug candidate to inhibit wear debris-induced periprosthetic tissue inflammatory osteolysis.

Bone marrow mesenchymal stem cells and osteoprogenitors are the precursors of osteoblasts. The reaction of these cells to wear particles is critical to both initial osseointegration of implants and long-term formation of the periprosthetic bone. Recent studies have shown that wear particles also contribute to osteolysis by damaging the osteogenic potential of osteoprogenitors and mesenchymal stem cells [3]. For example, polymethylmethacrylate (PMMA) particles have been previously shown to inhibit the osteogenic differentiation both in the bone marrow stromal cells [13] and in murine MC3T3 osteoprogenitor cells [14]. Because bone remodeling relies on a delicate balance between osteoblastic bone formation and osteoclastic bone resorption, an inhibitory effect on osteoblast production can tilt the balance in the direction of accelerated osteolysis.

Though we have shown that EM has inhibitory effects on osteoclast activity [9, 11], the potential effects of EM on osteoblast growth and differentiation are still not very clear. The objective of this study was to investigate whether treatment with EM can diminish the inhibitory effects of PMMA particles on MC3T3-E1 osteoprogenitor cells in vitro.

## 2. Material and Methods

### 2.1. Osteoblast Culture

Mouse MC3T3-E1 preosteoblasts (ATCC, CRL-2593) were cultured in *α*-modified minimum essential medium (*α*-MEM; Cellgro, VA) containing 10% heat-inactivated fetal bovine serum (FBS; Cellgro, VA) and antibiotics (100 units/mL of penicillin-G and 100 *μ*g/mL streptomycin) at 37°C in a humidified incubator with 5% CO_2_. For the cell differentiation experiment, MC3T3 cells were cultured in osteogenic media (regular media described above plus 10 mM *β*-glycerophosphate and 50 *μ*g/mL L-ascorbic acid (Sigma)) before they were harvested at 7 and 14 days, respectively. Culture media were changed every three days.

### 2.2. PMMA Particle Preparation

PMMA particles with mean diameter 6 *μ*m (range 0.1–10 *μ*m) were purchased from a commercial resource (Polysciences, Warrington, PA). Endotoxin was removed from the particles according to the protocol described by Ragab et al. [15]. The particles were rinsed in ethanol four times and sterilized in 70% ethanol with shaking overnight. The particles were then rinsed four times with PBS and resuspended in serum-free *α*-MEM. The removal of endotoxin was confirmed by a Limulus amoebocyte lysate assay (Cat number QCL-1000; BioWhittaker, Walkersville, MD) with sensitivity of 0.005 endotoxin unit (EU)/mL.

### 2.3. Erythromycin (EM) Treatment

EM was purchased from Sigma (Cat number E-5389). Stock solution was prepared following manufacturer's instruction for the cell culture experiments. We dissolved 10 mg EM powder into 20 *μ*L 100% ethanol, followed by the addition of distilled water up to 10 mL (final concentration 1 *μ*g/*μ*L). The stock solution was filtered prior to use. For the EM experiment, MC3T3 cells were pretreated with EM at different concentrations (0, 0.2, 1, 5, and 10 *μ*g/mL) for three hours before adding PMMA particles. MC3T3 cells without PMMA treatment were included as controls. Culture media and drugs were replaced every three days until the predetermined dates. The reason we pretreated cells with EM prior to addition of PMMA particles was to avoid the insufficient interaction of EM with cells because of the binding of tiny amount of EM to the PMMA particles [16]. Using the same experiment approach, we demonstrated that EM pretreatment resulted in a dose-dependent inhibition of NF-*κ*B activity in PMMA particles-activated murine RAW264.7 monocyte cells [9].

We found that EM, at the concentration up to 10 *μ*g/mL, is nontoxic to MC3T3 cells under physiological condition. In a separate study, we investigated the effect of EM on osteoblast differentiation and osteogenic gene expression in the absence of PMMA particles.

### 2.4. Lactate Dehydrogenase (LDH) Activity

Cell toxicity was determined by measuring the LDH release from dead or dying cells into the culture medium by applying a colorimetric method following the manufacturer's instruction (Roche Diagnostics BmbH). Culture medium was collected at 24, 48, and 72 hrs after EM treatment at different concentrations in PMMA-challenged cells. Then 100 *μ*L of culture medium was added into a plate of 96 wells, mixed with 100 *μ*L working solution, and incubated at room temperature for 30 minutes in the dark. Then, the plate was read under the UVmax colorimeter (molecular devices) at OD 490 nm. Blank culture medium was used as a control. LDH activity was expressed as absorbance (OD) per mg protein. This experiment was repeated three times and each experiment has duplicate samples.

### 2.5. Cell Counts

For cell proliferation assay, MC3T3 cells were plated in 12-well plates at an initial concentration of 1.5 × 10^5^ cells/well. Cells were pretreated with EM at different concentrations (0–10 *μ*g/mL) for three hours, followed by addition of PMMA particles (1 mg/mL, equal to around 5 × 10^7^ particles/mL). Cells were isolated by trypsinization from culture plates at 24, 48, and 72 hours of culture, respectively. Isolated cells were washed and cell number and viability were measured with a hemocytometer using trypan blue dye exclusion test. Direct cell counts were performed in duplicate. The experiments were repeated three times (each with duplicate samples).

### 2.6. Measurement of Cellular ALP Activity

For cell differentiation assay, MC3T3 cells were plated in 24-well plates at an initial concentration of 5 × 10^4^ cells/well. Cells were pretreated with EM at different concentrations (0–10 *μ*g/mL) for three hours, followed by addition of PMMA particles (1 mg/mL, equal to around 5 × 10^7^ particles/mL). Fourteen days after culture of MC3T3-E1 cells in the osteogenic medium, media were removed and the cell monolayer was gently washed twice with ice-cold phosphate buffered saline (PBS). Cell lysates were homogenized with the buffer from the ALP assay kit (BioVision, cat number K412-500, San Francisco, USA), followed by centrifugation (14,000 ×g for 5 min) to remove insoluble material. ALP activity in cell lysate samples was measured at OD 405 nm (UVmax colorimeter, molecular devices). ALP activity was expressed in nanomole of p-nitrophenol produced per minute per microgram of protein. All experiments were conducted in duplicate and repeated in three independent experiments.

### 2.7. Real-Time Quantitative Polymerase Chain Reaction (RT-PCR)

MC3T3 cells were cultured in osteogenic medium for 7 days in 12-well plates at an initial concentration of 1.5 × 10^5^ cells/well. Cells were pretreated with EM (0, 0.2, 1, 5, and 10 *μ*g/mL) for three hours before adding PMMA particles (1 mg/mL). Total RNA from cultured cells was reverse transcribed to cDNA as described elsewhere [17]. Real-time quantitative PCR was carried out according to the manufacturer's instruction (Perkin Elmer-Applied Biosystems, Foster City, CA). Primers used in this study were as follows: NF-*κ*B, 5′-GGGGATGTGAAGATGTTG-3′ (forward), and 5′-CCAAGTGCAGAGGTGTCTGA-3′ (reverse); Runx2, 5′-TGCTTCATTCGCCTCACAAA-3′ (forward), and 5′-TTGCAGTCTTCCTGGAGAAAGTT-3 (reverse); osterix, 5′-CCTCTCG  ACCCGACTGCAGATC-3′ (forward), and 5′-AGCTGCAAGCTCTCTGTAACCATGAC-3 (reverse); osteocalcin, 5′-AGGGAGGATCAAGTCCCG-3′ (forward), and 5′-GAACAG ACTCCGGCGCTA-3′ (reverse). To standardize the target gene level with respect to variations in RNA and cDNA, the housekeeping gene GADPH was used as an internal control. To determine the relative level of gene expression, the comparative C_T_ (threshold cycle) method with arithmetic formulae was used. Subtracting the C_T_ of the housekeeping gene from the C_T_ of target gene yielded the ΔC_T_ for each group (control and experimental groups), which was entered into the equation 2^−ΔC_T_^ and calculated for the exponential amplification of PCR. The gene activity in control group (PBS) was arbitrarily assigned to 1 to serve as a reference. The expression of the target gene from experimental groups therefore represents the fold-difference expression relative to the reference gene expression.

### 2.8. Statistical Analysis

The results were evaluated by the statistical test analysis using the ANOVA test, with the Schafer formula for* post hoc* multiple comparisons, using the SPSS software package (version 7.5; SPSS Inc., Chicago, IL). All treatments were repeated three times, each with duplicate samples, except RT-PCR (triplicate samples). Data was expressed as mean ± standard error of the mean. A *P* value of less than 0.05 was considered as significant difference.

## 3. Results

### 3.1. Effects of EM on Viability of PMMA-Challenged MC3T3-E1 Cells

**Figure 1:**
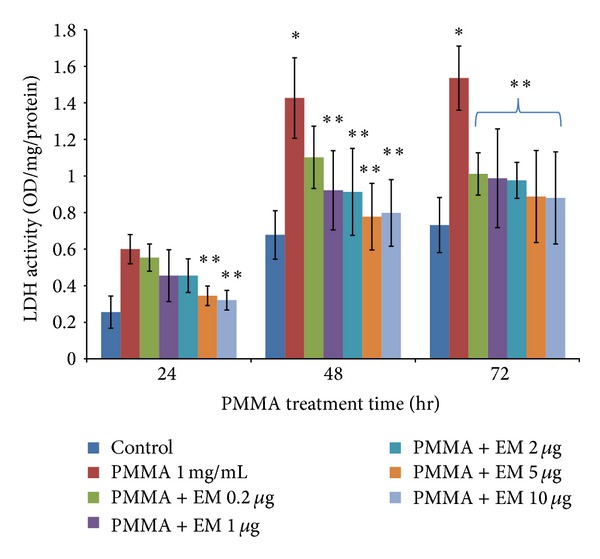
Effect of EM on the LDH release from PMMA-challenged MC3T3 cells into the medium. The values shown are means ± SEM from three experiments, each with duplicate samples, *P < 0.05 versus control (cell alone), **P < 0.05 for PMMA with EM treatment versus PMMA without EM treatment.

**Figure 2:**
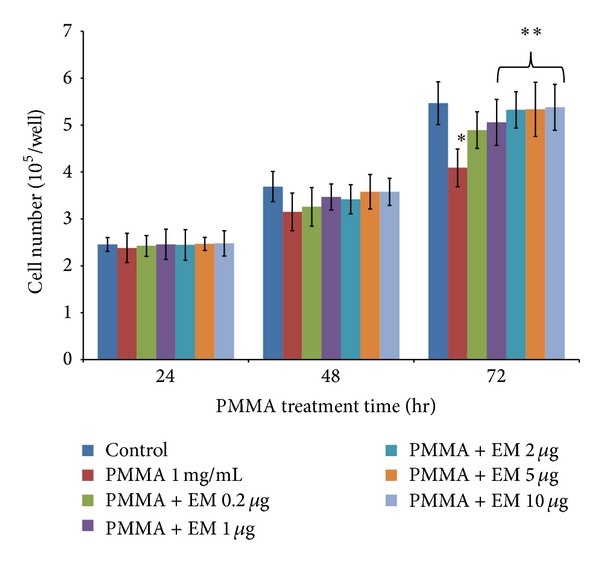
Proliferation of MC3T3 cells as measured by direct cell counting. Each bar denotes the mean ± SEM from three experiments, each with duplicate samples, *P < 0.05 versus control (cell alone), **P < 0.05 for PMMA with EM treatment versus PMMA without EM treatment.

Addition of PMMA particles (1 mg/mL) to MC3T3 cells in nonosteogenic culture resulted in a time-dependent increase in LDH release over a 72 h period, compared with untreated cells. PMMA particle-induced increase of LDH release was evident at 48 h (*P* = 0.075) and more significant at 72 h (Figure 1, *P* < 0.05). EM treatment attenuated PMMA-induced LDH release in a dose-dependent manner. EM concentrations higher than 5 *μ*g/mL (at 24 hours) or higher than 1 *μ*g/mL (at 48 hours) were required to significantly reduce LDH release. These data indicated that EM treatment mitigates PMMA particle-induced MC3T3 cell damage (necrotic or apoptotic change). The number of viable adherent cells was remarkably reduced after PMMA treatment at the time of 72 h (Figure 2, *P* < 0.05). Compared to EM-untreated cells (but PMMA-treated), the numbers of viable cells were slightly increased after EM treatment during the time of 24 and 48 h, which was not statistically significant. An increase of viable cells was found at the time of 72 h with the addition of EM at concentrations of 2, 5, and 10 *μ*g, respectively (*P* < 0.05 versus PMMA without EM treatment).

### 3.2. Effects of EM on ALP Activity of PMMA-Challenged MC3T3 Cells

**Figure 3:**
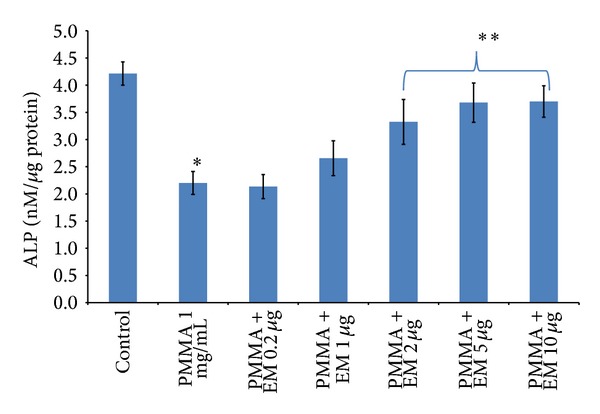
Effects of EM on intracellular ALP activity in MC3T3 cells. PMMA-challenged MC3T3 cells were cultured in 24-well plate at 2 × 10^5^ cells/well. Cells were treated with EM (0–10 *μ*g/mL) for 14 days. This assay was performed three times, each with duplicate samples. *P < 0.05 versus control (cell alone); **P < 0.05 for PMMA with EM treatment versus PMMA without EM treatment.

To determine the effect of EM on osteoblast function, intracellular ALP activity was measured after 14 days in culture. PMMA treatment significantly reduced the intracellular ALP activity by 49% at day 14, compared to controls (Figure 3, *P* < 0.05). In the presence of PMMA, pretreatment with EM at the range of 2–10 *μ*g/mL significantly elevated intracellular ALP activity in a dose-dependent manner (*P* < 0.05).

### 3.3. Effects of EM on Osteogenic Gene Expression of PMMA-Challenged MC3T3 Cells (Figure 4)

MC3T3 cells exposed to PMMA particles showed a significant reduction in gene expression of Runx2 (*P* < 0.05) and osterix (*P* < 0.05). EM treatment (2–10 *μ*g/mL) partially restored the gene expression of Runx2 and osterix reduced by PMMA treatment (*P* < 0.05). In addition to the transcription factors, PMMA decreased the expression of osteocalcin, a known marker for osteoblast differentiation (*P* < 0.05). EM treatment significantly increased the osteocalcin gene expression in a dose-dependent manner (*P* < 0.05). It should be noted that EM could not reverse the gene expression of Runx2, osterix, and osteocalcin to the level that was similar to the controls. Gene expression of NF-*κ*B, however, was significantly increased after PMMA particle exposure (2.8-fold, *P* < 0.05). EM treatment inhibited PMMA-stimulated NF-*κ*B gene expression at the concentration of 2–10 *μ*g/mL (*P* < 0.05).

### 3.4. Effects of EM on ALP Activity and Osteogenic Gene Expression under Physiological Condition (in the Absence of PMMA Stimuli)

We designed separate experiments to determine whether EM has effects on the differentiation and gene expression of MC3T3 cells under physiological condition (without PMMA stimuli). As shown in Figure 5(a), there is no significant difference of ALP activity between cells without and with EM treatment after 14 days in culture. There are no significant differences in MC3T3 cells in osteogenic gene expression between cells treated with and without EM (Figure 5(b)).

## 4. Discussion

In vivo bone turnover is determined by a delicate balance between osteoclastic bone resorption and osteoblastic bone formation. Although activated macrophages and enhanced osteoclast activity are integral elements underlying bone destruction in periprosthetic osteolysis [18, 19], the effects of wear particles on osteoblastic cells significantly reduce the replacement of bone at the prosthetic interface [3]. Recent studies revealed that wear particles profoundly alter the proliferation, maturation, and differentiation of osteoprogenitors, thereby contributing to the osteolytic process by decreasing bone formation [3, 14, 20]. Many types of orthopaedic wear particles can affect osteoblast viability, proliferation, and function, such as titanium particles [21], ultra-high-molecular weight polyethylene (UHMWPE) [20], and PMMA [14]. This study revealed that EM treatment significantly diminished the inhibitory effects of PMMA particles on cell proliferation, viability, and differentiation of murine MC3T3 osteoprecursor cells. Though the detailed molecular mechanism behind this action is unclear, it might be due to the downregulation of PMMA-induced activation of NF-*κ*B signaling.

Previous studies have shown that the proliferation and viability of mature osteoblasts and osteoblast precursors are impaired by wear debris, which occur after particle phagocytosis and are accompanied by the disruption of intracellular cytoskeletal structures and organelles [3, 20]. Our data demonstrated that cellular viability was impaired after exposure to PMMA particles, as evidenced by the increase in LDH release over time (Figure 1) and the reduction in cell number (Figure 2) over 72 hrs. Pretreatment with EM diminished inhibitory effect caused by PMMA particle on cell viability. In a separate study, we notice that EM (up to 10 *μ*g/mL) has no cytotoxic effects in MC3T3 cells under physiological condition (data not shown), providing further evidence that EM represents a selective osteoclast inhibitor and has little effect on osteoblast biology under physiological condition. Instead, EM mitigates the catabolic effect of MC3T3 cells exposed to PMMA particles.

Chiu et al. [14] reported that PMMA particles inhibited the ALP activity of MC3T3 cells in a dose-dependent manner. In this study, we found that PMMA (1 mg/mL) significantly inhibited ALP activity up to 50% (Figure 3). EM significantly attenuated the reduction of ALP production by PMMA particles in MC3T3 cells. Osteocalcin, also known as bone Gla protein, is the most abundant noncollagenous protein of bone and a marker of terminal osteoblast differentiation [22]. Our data demonstrated that EM increased the gene expression of osteocalcin, suggesting that EM had a mitigative effect on PMMA-induced reduction of osteocalcin gene expression.

Osteoblast differentiation is regulated by a panel of transcription factors such as Runx2 and osterix [23]. Runx2 regulates the expression of key osteoblast proteins such as ALP and osteocalcin. Osterix lies downstream of Runx2 and is required for the differentiation of preosteoblasts into mature osteoblasts [23]. Chiu et al. [24] reported that the gene expression of Runx2 and osterix in MC3T3-E1 cells is reduced after PMMA treatment. The mechanism of PMMA particles action on osteoblasts is not very clear. Ma et al. [25] found that activation of p38 mitogen-activated protein kinase (MAPK) is required for osteoblast differentiation. When treated with PMMA particles, differentiating MC3T3-E1 cells demonstrates significant suppression of p38 activity and transforming growth factor- (TGF-) b1, which is involved in osteoblast differentiation. These data indicate that the disrupting upstream signaling pathways, receptor-matrix interactions, or cellular synthesis and transport mechanisms might be involved in the development of PMMA particle- induced osteoblast dysfunction. We found that EM treatment prevents the decrease in the gene expression of Runx2 and osterix in MC3T3 cells treated with PMMA particles.

**Figure 4:**
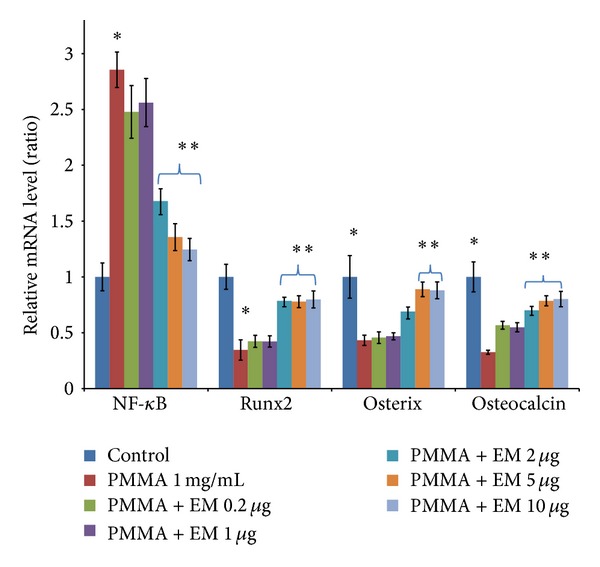
Effects of EM on gene expression of NF-*κ*B, Runx2, osterix, and osteocalcinin MC3T3 cells 7 days in culture. An increase of NF-*κ*B (2.8-fold) was observed in MC3T3 cells challenged with PMMA particles, compared to PBS control. This increase was significantly reduced by EM treatment, as determined by ANOVA analysis. Values are means ± SEM of genes measured for triplicate and the experiments repeated three times. *P < 0.05 cells + PMMA versus control (cell alone); **P < 0.05 for PMMA with EM treatment versus PMMA without EM treatment.

**Figure 5:**
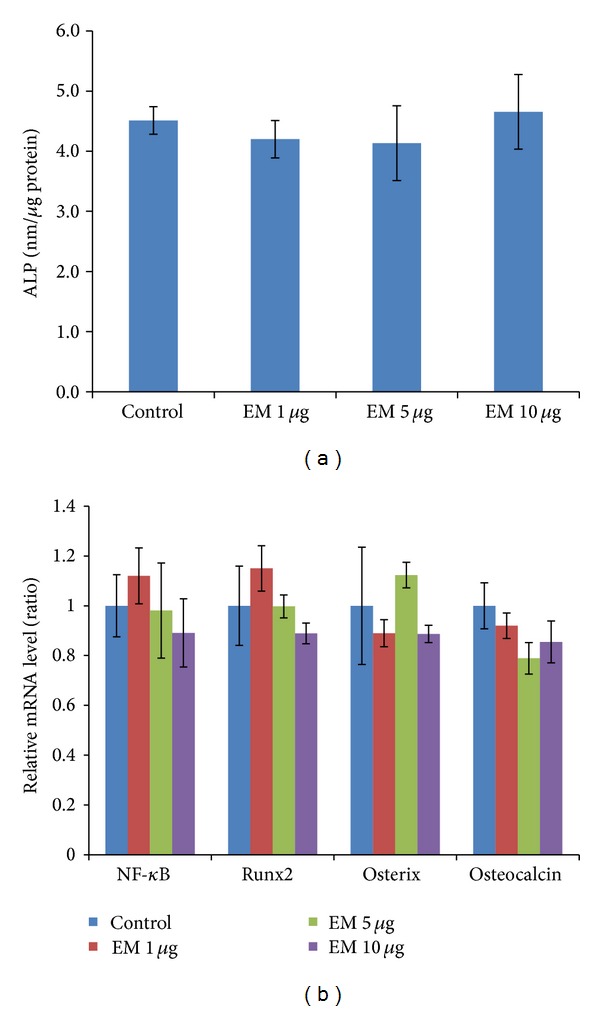
Effects of EM on ALP activity and osteogenic gene expression in MC3T3 cells under physiological condition. (a) MC3T3 cells were cultured in 24-well plate at 2 × 10^5^ cells/well. Cells were treated with EM (0–10 *μ*g/mL) for 14 days before ALP activity assay. Experiments repeated three times, each with duplicate samples. (b) Gene expression of MC3T3 cells 7 days after culture. Values are means ± SEM of genes measured for triplicate, and the experiments repeated three times.

Although the actions of the transcription factor NF-*κ*B on osteoclastogenesis are well known [26], the potential actions of NF-*κ*B on osteoblasts and bone formation are still not very clear. Our data revealed that PMMA particles significantly increased NF-*κ*B gene expression (Figure 4). The mechanism of PMMA-induced NF-*κ*B activation might be multifactorial. There is compelling evidence that proinflammatory cytokines, such as TNF*α* and IL-1*β*, inhibit osteoblast differentiation and bone formation [27, 28], in part through the activation of NF-*κ*B pathway in osteoblast and its precursor cells [29, 30]. We revealed that EM treatment inhibits PMMA particle-induced increase of NF-*κ*B gene expression in MC3T3 cells at the concentration of 2 *μ*g/mL or higher (Figure 4). We propose that EM counters the inhibitory effects of PMMA on MC3T3 Runx2 and osterix expression and hence partially restores the ALP activity, through downregulation of NF-*κ*B activity. Inflammatory cytokines and wear debris reduction in Runx2 gene expression could be through both enhancement of Runx2 degradation [31] and activation of NF-*κ*B pathway, because Runx2 promoter contains a homologous sequence of NF-*κ*B and activated protein-1 (AP-1) binding sites that are capable of conferring responsiveness to cytokines and wear debris [32, 33]. Overexpression of NF-*κ*B is associated with the downregulation of Runx2 gene expression [27]. These findings, including the data reported here, are of particular interest based on our recent finding in a rat model of revision orthopaedic implantation [34]. We found periprosthetic EM delivery significantly inhibits wear debris induced tissue inflammation. Improvement of local tissue inflammation was accompanied by a reduction in osteolysis and an increase in new bone formation. This could be the result of either increased new bone formation or reduced osteolysis after EM treatment.

We propose that reduction of inflammation minimizes the inhibitory effects of inflammatory cytokines on osteoblast and osteoblast precursors, in part by diminishing the activation of NF-*κ*B signaling. More efforts are required to clarify the interplay between NF-*κ*B signaling and Runx2 signaling in osteoblast and osteoblast precursors. It should be noted that our data was based on a murine osteoblast precursor cell line and it may not reflect the effects of EM in a more clinical relevant environment in which osteoblast and osteoclast interaction occurs. Future studies should include a simultaneous evaluation of cellular activity and signaling using coculture systems of osteoblast and osteoclast under both physiological and inflammatory conditions. In addition, inhibition of NF-*κ*B gene expression by EM treatment in PMMA-challenged MC3T3 cells leads to a partial restoration of cell biological activity, indicating other unknown transcriptional regulation might be also involved in the regulation of osteoblast function after PMMA treatment.

In conclusion, our data indicated that EM mitigates the inhibitory effects of PMMA on MC3T3 cell viability and differentiation, in part through downregulation of NF-*κ*B pathway and partial restoration of the gene expression of Runx2 and osterix. EM appeared to represent an anabolic agent on MC3T3 cells challenged with PMMA particles, while preserving its selective inhibition of osteoclast activity. Considering the increased clinical use of cementless joint replacements, further studies are necessary to determine whether EM has similar effects on other wear particles-induced dysfunctional changes of osteoblast and osteoblast precursors. We propose that periprosthetic EM delivery may represent a new therapeutic candidate for the prevention and treatment of wear debris-associated osteolysis and aseptic loosening.
